# Rare 48, XYYY syndrome: case report and review of the literature

**DOI:** 10.1002/ccr3.1311

**Published:** 2017-12-07

**Authors:** Maryam Abedi, Arash Salmaninejad, Ebrahim Sakhinia

**Affiliations:** ^1^ Department of Animal Science Faculty of Natural Sciences Tabriz University Tabriz Iran; ^2^ Drug Applied Research Center Student Research Committee Tabriz University of Medical Sciences Tabriz Iran; ^3^ Medical Genetics Research Center Student Research Committee Department of Medical Genetics Faculty of Medicine Mashhad University of Medical Sciences Mashhad Iran; ^4^ Connective Tissue Research Center Department of Medical Genetics Faculty of Medicine and Tabriz Genetic Analysis Centre (TGAC) Tabriz University of Medical Sciences Tabriz Iran

**Keywords:** 48, XYYY syndrome, chromosomal abnormality, cytogenetic, QF‐PCR, sex chromosome

## Abstract

48, XYYY syndrome is a rare condition. A male with 32‐year‐old and three Y chromosomes is described. This syndrome is phenotypically similar to Klinefelter syndrome. In this patient, Semi‐Klinefelter characteristics such as tall stature, teeth dysmorphology, long length of fingers, partial deformity of the joints, likewise mental health problems were obvious.

## Introduction

Sex chromosome aneuploidies (SCA) happen in approximately 1 in 400 live births 1. The most common abnormalities are 47, XXY (Klinefelter syndrome), 47, (Triple X), 47, XYY, and 45, X (Turner syndrome). The prevalence of males with more than one extra sex chromosome (e.g., 48, XXYY or 48, Y) is less frequent 2. Up to now, the psychiatric aspects of these syndromes have not been determined precisely. 48, XYYY and 49, XYYYY karyotypes are very rare: Less than ten cases have been described for 48, XYYY, and only three of them have been associated with a nonmosaic form 3, 4.

The first incidence of 48, XYYY sex chromosome aneuploidy was described in 1965 by Townes et al. as a rare type of sex chromosomes abnormality (48, XYYY). This syndrome is phenotypically similar to Klinefelter syndrome in many aspects 5. The patient described here was referred to the Tabriz Genetic Analysis Center complaining infertility. After a comprehensive genetic counseling, rapid sex chromosome counting via karyotype analysis and QF‐PCR was proposed for him. QF‐PCR outcomes indicated an extra pair of Y chromosomes and karyotyping confirmed 48, XYYY syndrome.

## Case Presentation

The patient was 32 years old when he was referred to our center, and he was complaining of infertility. Pregnancy and delivery were normal. He was born at term with standard measurements. On physical examination, his weight was 74 kg, his height was 190 cm, and also body mass index (BMI) was normal at 20.5 (20 < normal values < 25). In clinical examination, Semi‐Klinefelter characteristics such as tall stature (190 cm), teeth dysmorphology, history of respiratory disease, low total body hair, long length of fingers and toes, partial deformity of the joints and nails, likewise mental health problems were obvious in this patient. Meanwhile, there was no similar features or history reported within the patient's family members. After consultation, we decided to accomplish karyotype analysis to make sure about chromosome numbers and aberrations that was needed for early diagnosis and then choose appropriate treatment strategies.

The patient's blood samples were cultured, and their chromosomes were analyzed and studied by the G‐banding method. A total of 85 cells were counted of which 81 cells had 48 chromosomes, two had 46 and two had 47.

All of the 85 counted cells without exception illustrated three Y sex chromosomes (the cells containing 46 and 47 chromosomes were probably caused by random chromosomal additions or losses from cell breakage). The construction of the cells with 48 chromosomes is thought to be 48, XYYY. The supplementary chromosomes were Y chromosomes because they had the subsequent characteristics in common: The long arms of the chromatids usually remained equal through their length, they never contributed in satellite association, and almost always appeared on or around the border of the metaphase spread, and simultaneously there are no clinical signs attributable to aneuploidy of other G group members.

**Figure 1:**
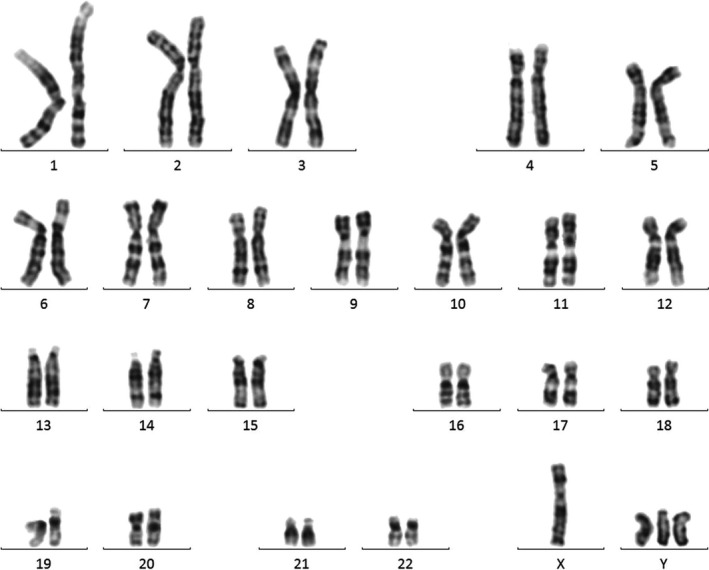
Peripheral blood karyotype result belonging to the subject: compatible with 48, XYYY.

**Figure 2:**
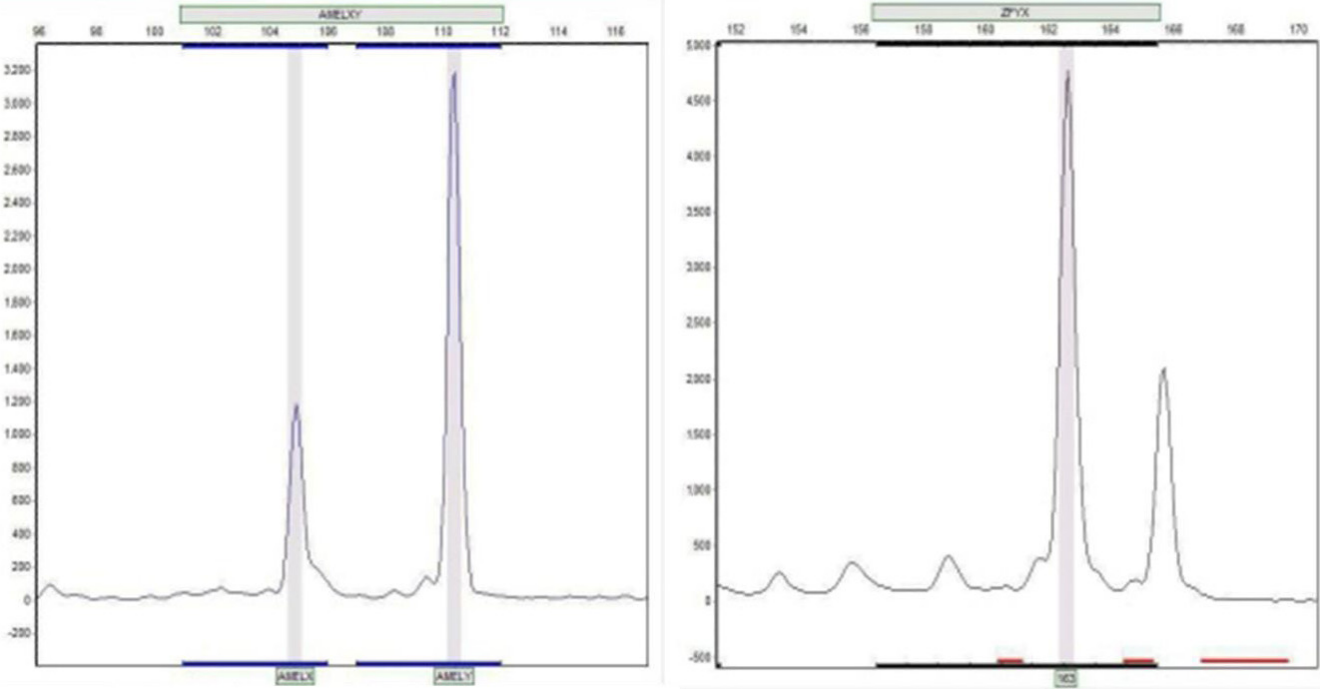
QF‐PCR result indicates extra pair of Y chromosomes. The AMELXY marker amplifies nonpolymorphic sequences on the X (AMELX) and Y (AMELY) chromosomes and can be used to determine the presence or absence of a Y chromosome. AMELXY may be used to assess the relative number of X to Y chromosomes. The ZFYX marker is a nonpolymorphic (non‐STR) marker present on both the X and Y chromosomes. These markers may be used to assess the total number of sex chromosomes when informative. It is not possible to determine which allele represents the X or Y chromosome.

His karyotype was therefore reported as 48, XYYY (Fig. 1). Cytogenetic examination of his wife was normal (46, XX). In order to validate our results, we used QF‐PCR, of which the results implied the existence of three Y chromosomes in this patient (Fig. 2). Complete blood count, liver function tests, renal function tests, thyroid function test, hormones test, and endocrinological profile were found to be normal also spermogram of the patient indicated absolute azoospermia. Informed consent was obtained from the patient for publication of the clinical findings and laboratory test results.

## Discussion

While the incidence of Klinefelter syndrome is one in 650 male births, the incidence of 48, XYYY syndrome is incredibly rare and approximately 10 cases have been reported up to date, including mosaicisms. That is why there is very little phenotypic data on this syndrome. The case presented here is the first case reported from Iran and 7th of the world 6.

The patient seemed to have particular communication impairments and social interplay that could be defined by cognitive problems. In a study evaluating the risk of social behavior disorder in 62 males with SCA (20 XXY, 22 XYY, and 20 XXYY gonosomal systems), none of 47, XXY, 36% of 47, XYY, and 50% of 48, XXYY patients were diagnosed with autism spectrum disorder (ASD) according to the SCQ and ADOS‐G 7, 8.

There are limited documented cases of male patients with XYYY and XYYYY syndromes. These syndromes result from Y chromosome nondisjunction through spermatogonial mitosis related to nondisjunction in meiosis or a simple sequence of events would include nondisjunction of the Y chromosome in two early divisions of an XY zygote. Whole descriptions in the literature have reported persons with behavioral disturbances, but the characterization of the psychiatric phenotype remains restricted 9.

An XYYY genotype may result from the fertilization of a normal ovum by a YYY sperm. A sperm comprising three Y chromosomes can hypothetically be produced by anaphase lagging or secondary nondisjunction in meiosis II from an XYY primary spermatocyte. The father of our propositus may have 47, XYY genotype, but this is considered improbable since he presented none of the phenotypic characteristics frequently accompanying with the possession of a supernumerary Y chromosome. There is, nevertheless, the likelihood that his testicular tissue alone was 47, XYY. Also, recurrent nondisjunction of the Y chromosome in an ordinary zygote may be probable to yield a mosaic of at least three cell lines, X, XY, and XYYY. In this regard, a familial propensity to nondisjunction has been explained 5, 10. In this presented case, no evidence to support either a mosaic in the chromosome analysis of the propositus or clinical abnormalities in his family was detected.

The mechanism by which this patient received his extra Y chromosomes is not clear. The most likely explanation appears to nondisjunction in spermatogonial mitosis followed by a 2nd nondisjunction of one of the Y chromosomes in meiosis consequential in the formation of a sperm bearing 3 Y chromosomes.

The triple Y condition appears compatible with a reasonable life span, and this is in agreement with the proposition that the Y chromosome contains little important genetic material other than that responsible for testicular formation 11. Two supernumerary Y chromosomes are apparently attended by sterility. Major phenotypic defects are not expected due to the low genetic density of the Y chromosome 12. The patient showed some clinical features similar to those of Klinefelter syndrome. This case represents the 7th instance described in the literature of a 48, XYYY karyotype without detectable mosaicism. In Table 1 and Table 2, the authors gathered developmental features and medical concerns, respectively, from those of the previously reported cases also, and all of the previous case reports about this syndrome are listed in Table 3.

**Table 1:** Developmental features related to 48, XYYY syndrome

Development				
Growth	Learning	Mobility and activity	Speech and communication	Behavior
Birth weight is normal	Mild learning difficulties	Slight delay in sitting, walking, and control of body movements	Speech delay	Impulsivity and a low frustration tolerance
	IQ range is low normal (65–86)		Speech difficulties	Prone to aggressive outbursts
Stature is usually tall with normal average height	Academic skills have been quite variable	Low muscle tone	Ability to live independently	Low emotional stability
	Raised performance/verbal IQ ratio	Unusual and loose joints		Vulnerability to behavioral and social difficulties

**Table 2:** Some most important medical concerns about 48, XYYY syndrome

Medical concerns		
Chest infections, asthma, coughs, and colds	Teeth	Other features
Upper respiratory infections and coughs in childhood	Poor enamel formation and discoloration	Compatible with a reasonable life span
		Flat feet or bony foot deformities
		Minor kidney anomalies
Asthma and atopy in adulthood	Irregular or very large teeth	Sterility
		Radioulnar synostosis
		Acne

In our case, no evidence to support either a mosaic in the chromosome analysis of the propositus or clinical abnormalities in his family was detected.

**Table 3:** Previous studies reported 48, XYYY syndrome (with/without mosaicism)

Publication year	Authors	Condition	Ref
1965	Townes PL, Ziegler NA, Lenhard LW.	48, XYYY	5
1967	D Cox, C L Berry	Mos45, X/48, XYYY	9
1972	Schoepflin GS, Centerwall WR.	48, XYYY	13
1973	Hunter H, Quaife R.	48, XYYY	10
1975	Sele B, Bachelot Y, Richard J, Muller J, Jalbert P, Berthet J.	Mos46, XX/47, XYY/48, XYYY	14
1980	Gigliani F, Gabellini P, Marcucci L, Petrinelli P, Antonelli A	Mos47, XYY/48, XYYY/49, XYYYY	8
1988	Hori N, Kato T, Sugimura Y, Tajima K, Tochigi H, Kawamura J.	48, XYYY	16
1989	Bryke CR, Mahoney MJ, Yang‐Feng TL.	Mos45, X/48, XYYY (prenatal diagnosis)	17
1991	Mazauric‐Stüker M, Kordt G, Brodersen D	48, XYYY	18
1993	Teyssier M, Pousset G	Mos46, XY/48, XYYY	19
1994	Stein A, Heilbronner H, Jungmann J	48, XYYY	20
1995	James C, Robson L, Jackson J, Smith A	Mos46, XY/47, XYY/48, XYYY	21
1995	Fox JE, Blumenthal D, Brock W, Kreitzer P, Cooper R, Anderson D, Pleak R, Ehrenfreund L, Freedman S, Zaslav AL	Mos45, X/46, XY/48, XYYY	22
2002	Venkataraman G, Craft I	48, XYYY (in ICSI treatment)	23

Karyotyping is a slow and classical method of detecting extra chromosomes but its results produces trustworthy outcomes 24. In contrast to karyotyping, new modern methods of molecular cytogenetics such as QF‐PCR are rapid procedures with high accuracy to detect possible chromosomal aneuploidy. In QF‐PCR method, specific repeated DNA sequences of the chromosome known as small tandem repeats (STRs) are amplified by PCR. Using fluorescently labeled primers, visualization and quantification of the fluorescently labeled PCR products can be performed. Quantification can be achieved by calculating the ratio of the specific peak areas of the respective STR using an automated DNA sequencer. STRs vary in size between subjects, depending on the number tetra repeats present on each allele. DNA is amplified from normal subjects which are heterozygous (have alleles of different lengths) for a specific STR marker and expected to show two peaks of different length with the same peak areas. STR markers that are heterozygous are considered to be informative. DNA amplified from subjects who are trisomy will exhibit either three peaks with similar area, or only two peaks, one of them with the twice as large area as the other.

The AMELXY marker amplifies nonpolymorphic sequences on the X (AMELX) and Y (AMELY) chromosomes, and also this marker can be used to determine the presence or absence of a Y chromosome; likewise, it may be used to assess the relative number of X to Y chromosomes. The ZFYX marker is a nonpolymorphic (non‐STR) marker present on both the X and Y chromosomes. This marker may be used to assess the total number of sex chromosomes when informative.

QF‐PCR is based on polymorph‐none‐polymorph STRs which indicate the total number of chromosomes. The other advantage in QF‐PCR is related to allelic and none allelic STRs which may be useful for distinguishing the origin of a chromosome 25. A literature review revealed that individuals with this karyotype are infertile and have extensively varying phenotypes with regard not only to physical signs but also to brain functions and cognitive performance.

Additional informed consent was obtained from the patient for which identifying information is included in this article.

## Conflict of Interest

None declared.

## Authorship

All the authors: made substantial contribution to the preparation of this manuscript and approved the final version for submission. MA: designed and drafted the manuscript and prepared the images. AS: planned and wrote the manuscript; prepared the images; had important role to facilitate the work between different authors; and also did the literature review. ES: revised the manuscript for critically important intellectual content and approved for final submission.
